# Wearable Biomedical Measurement Systems for Assessment of Mental Stress of Combatants in Real Time

**DOI:** 10.3390/s140407120

**Published:** 2014-04-22

**Authors:** Fernando Seoane, Inmaculada Mohino-Herranz, Javier Ferreira, Lorena Alvarez, Ruben Buendia, David Ayllón, Cosme Llerena, Roberto Gil-Pita

**Affiliations:** 1 School of Engineering, University of Borås, SE-50190 Borås, Sweden; E-Mail: javierfg@kth.se; 2 School of Technology and Health, Royal Institute of Technology, SE-14152, Stockholm, Sweden; E-Mail: rubenbl@kth.se; 3 Department of Signal Theory and Communications, University of Alcala, ES-28871, Madrid, Spain; E-Mails: inmaculada.mohino@edu.uah.es (I.M.-H.); lorena.alvrezp@uah.es (L.A.); david.ayllon@uah.es (D.A.); cosme.llerena@uah.es (C.L.); roberto.gil@uah.es (R.G.-P.)

**Keywords:** bioimpedance, GSR, heart rate, mental stress, non-invasive measurements, textile electrodes, speech analysis, multimodal signal processing

## Abstract

The Spanish Ministry of Defense, through its Future Combatant program, has sought to develop technology aids with the aim of extending combatants' operational capabilities. Within this framework the ATREC project funded by the “Coincidente” program aims at analyzing diverse biometrics to assess by real time monitoring the stress levels of combatants. This project combines multidisciplinary disciplines and fields, including wearable instrumentation, textile technology, signal processing, pattern recognition and psychological analysis of the obtained information. In this work the ATREC project is described, including the different execution phases, the wearable biomedical measurement systems, the experimental setup, the biomedical signal analysis and speech processing performed. The preliminary results obtained from the data analysis collected during the first phase of the project are presented, indicating the good classification performance exhibited when using features obtained from electrocardiographic recordings and electrical bioimpedance measurements from the thorax. These results suggest that cardiac and respiration activity offer better biomarkers for assessment of stress than speech, galvanic skin response or skin temperature when recorded with wearable biomedical measurement systems.

## 1. Introduction

The Spanish Future Combatant Program (ComFut) developed by the Spanish Ministry of Defense pursues the integration of technology aids for the combatant with the aim of including not only intelligence facilities, geographical information systems or improved ballistics, but also systems able to analyze the physical and psychological state of the individual. With the aim of extending operational capability, the combatant must be not only by physically ready, but also mentally trained, which encompasses from stress management to dream deprivation. In this issue, information supported by augmented reality systems and real time monitoring of biometrics can be used to improve the mental capabilities of the future combatant.

In this framework, this paper presents the ATREC project funded by the “Coincidente” Spanish program of the Ministry of Defense, which aims at analyzing the use of biometrics and real time monitoring of combatant stress levels. This is a novel and multidisciplinary objective, since it includes the combination of wearable sensors, signal processing, pattern recognition and psychological analysis of the obtained information.

### 1.1. The ATREC Project

**Figure 1.**
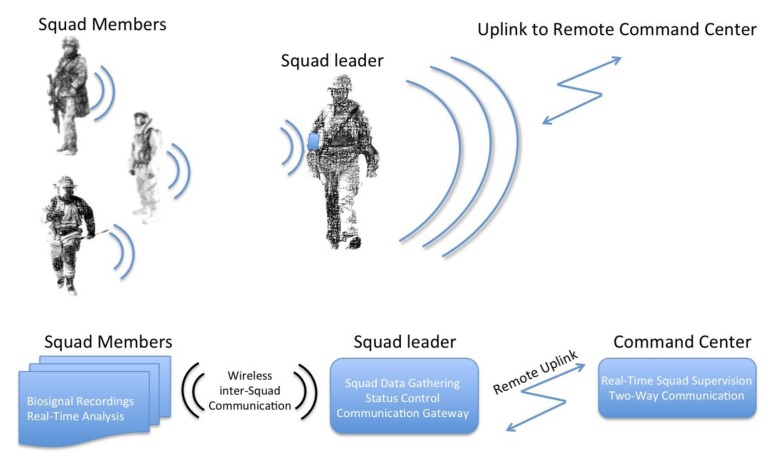
System overview of the full system aimed to be implemented during the ATREC project integrating sensorized garments, wireless communication and wearable computing for real-time assessment.

ATREC is the acronym for the Spanish project name “Analisis en Tiempo Real del Estrés del Combatiente” (Real-Time Analysis of the Stress of the Combatant). The main objective of the project is to set up a complete system to assess in real time the emotional, physical and mental stress load of a combatant during combat. It is based on the use of wearable measurement systems for the acquisition of non-invasive physiological measurements and wireless communication. Figure 1 shows a diagram of the whole system.

The project is divided in two main stages or phases. In a first phase, the ATREC project aims at identifying indicators suitable for monitoring Autonomous Nervous System (ANS) activity that can feasibly be acquired by customized wearable instrumentation. In this stage a set of sensorized garments has been implemented for performing non-invasive recording of several physiological variables. These variables include galvanic skin response (GSR), body temperature, electrocardiogram (ECG), thoracic electrical bioimpedance (TEB), and voice recording for speech analysis. In order to carry out this stage, a system prototype has been designed and implemented, and it has been used in a set of controlled stress experiments with a wide number of subjects. The prototype system is based on several different measurement systems that combined with different sensorized garments has allowed changes in the position and modalities of the different sensors, generating a wide number of possible measurement configurations.

In a second phase, a final prototype has been implemented allowing the measurement of the selected most important measurements, with the final configuration of the sensors. It consists in a uniform vest that embeds all the electronics and signal processing algorithms to assess and represent the different stress levels in both the soldier and command displays.

This manuscript is mainly focused on the work done during the first phase, the one targeting the selection of the main biomedical parameters for assessment of mental stress and workload. The second phase addresses the construction of sensorized vest and, its validation and the usability study is out of the scope of this manuscript and it will be described elsewhere.

### 1.2. Wearable Biomedical Measurement Systems

Advances in the fields of electronics and instrumentation technology, together with novel textile materials and novel textile-electronic integration technics have boosted the development and implementation of sensorized garments for wearable biomedical measurement systems [1], specially in the last decade.

As a result of extensive and numerous research efforts, several wearable and textile-enable monitoring systems for the performance of non-invasive measurements have been designed and implemented like a sensorized chest strap for cardiopulmonary activity status during emergencies [2], a sensorized vest incorporating fully woven textile sensors for monitoring ECG and respiration rate [3] or the HeartCycle Sensorized vest for detection of cardiogenic pulmonary edema [4]. A comprehensive listing of wearable biomedical measurement systems is available in [1].

Moreover, leaving aside research project prototypes, currently it is possible to find commercially available wearable sensing technologies for several different applications, ranging from simple wearable heart rate (HR) monitors for fitness, e.g., the Polar heart rate strap [5], to body worn multiparametric measurement systems, e.g., the EQ02 life monitor manufactured by Equivital [6] or the nECG shirt L1 developed by nuubo which is designed to measure ECG [7].

Available wearable biomedical measurement systems and the respective enabling technologies allow the development of solutions not only for personalized health monitoring applications e.g., management of chronic patients and home-care, but also for personal protective equipment for workers operating in dangerous and stressful conditions e.g., soldiers, firemen, bomb-squad members, *etc*.

### 1.3. Non-Invasive Assessment of Mental Stress

The brain and the spinal cord comprise the central nervous system (CNS), which through the Autonomic Nervous System (ANS) and peripheral innervation of organs and glands controls the heart's electrical activity, gland secretion, blood pressure, and respiration function among others to preserve the homeostasis of the organism, i.e., the human body in this case. The ANS is responsible for enabling and controlling the reaction of the body to external and internal stimuli. Such a control function is performed through the two branches of the ANS: The Sympathetic Nervous System (SNS) and the Parasympathetic Nervous System (PNS). Analysis of ANS activity regarding SNS and PNS is a common practice for assessing stress [8].

Evaluation of ANS activity can be performed by recording and analysing several physiological variables: heart rate [9], respiration rate (RespR) [10], ElectroEncephaloGraphical (EEG) activity [11], skin galvanic response (GSR) [12,13] and skin temperature [14].

The analysis of a speaker's speech can provide information about cognitive and affective task workloads as well as performance levels [15,16]. Interpersonal oral communication includes both verbal and paraverbal information that allows the communication of emotions and feelings [17]. Therefore, speech analysis if combined with the sensor-based detection process may improve the final performance of the stress detection system. In this line the aforesaid first phase of the ATREC project also includes the use of voice recordings for further speech analysis.

### 1.4. Sensorized Garments and Instrumentation for Assessment of ANS Activity

The available literature indicates that most of the wearable systems made for assessment of ANS activity have targeted the study of emotional, cognitive, physical arousal mental status and stress [12,18–28]. Nevertheless several wearable devices and sensorized garments have been implemented and used to record physiological variables to study the response of the ANS during stressful tasks in a non-invasive manner through, e.g., parameters from cardiac activity, GSR dynamics, skin temperature and RespR [10,13,29,30].

The sensorized garments manufactured for recording such relevant physiological variables are mostly chest straps [5,6,31–33] and t-shirts [7,20,21,34–40] aiming to record respiration and heart activity and gloves [20–23], glove-like devices [19,24,25] and wrist-bands [26–28,41] for recording GSR and temperature.

Since the turn of the century, sensorized gloves, gloves-like systems and wristbands have been used for obtaining GSR recordings and performing electrodermal response studies. With the passage of time the level of electronic integration has increased. Initially metallic [25–28] or Ag/AgCl external electrodes [20,21,24,41] were used, later a glove with an integrated electrodermal response sensor was manufactured [23], and an eventually gloves with textrodes integrated in the garment have been produced [22].

## 2. Materials

Several textile-enabled measurement devices and sensorized garments have been implemented for recording physiological variables that would allow to study the response of the ANS during stressful tasks in a non-invasive manner e.g., parameters from cardiac activity, GSR dynamics, skin temperature and RespR [10,13,29,30]. Some details relevant to the following Sections: Section 2.1 Measuring Devices and Section 2.2 Sensorized Garments have been published elsewhere [42,43] and validate the measurement performance of the wearable biomedical measurement system produced during the 1st phase of the ATREC project.

### 2.1. Wearable Biomedical Measurement Devices

For the 1st phase, two non-invasive physiological measurement devices have been built: One to measure GSR and skin temperature and a second device to record cardiogenic biopotentials and impedance thoracic measurements. Both are battery operated with 900 mAh lithium-ion batteries, and are provided with an internal 4GB SD memory card to allow long-term recording and off-line data analysis.

#### 2.1.1. GSR and Body Temp

**Figure 2.**
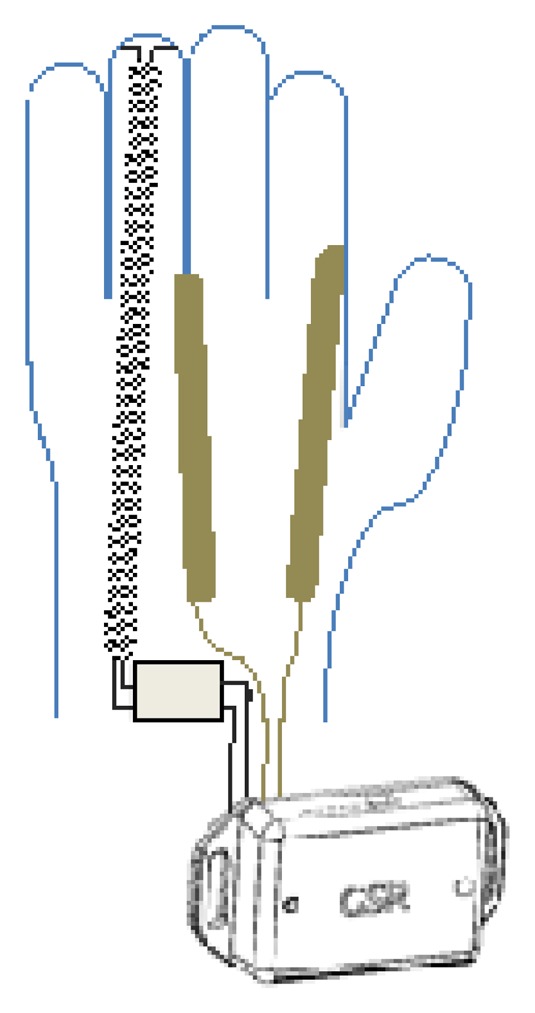
GSR device with a drawing of the connection with the sensorized glove.

One of the devices enables skin impedance monitoring as well as ambient and body temperature monitoring. The reduced dimensions (50 × 35 × 15 mm) enable its integration in a wearable biomedical measurement system in a straightforward manner. In this case, this device is intended to be used with the sensorized glove and/or the arm-strap that will be explained in the following Sections, see Figure 2.

For the GSR measurements a Wheatstone bridge topology with constant voltage excitation of 0.5 V is implemented. The voltage is applied to the skin through a resistor of 2 KΩ that limits the maximum current. The Wheatstone bridge differential voltage is measured by an instrumentation amplifier that it is connected to the input of an Analog-to-Digital Converter (ADC) available in the microcontroller in order to obtain the GSR value. The GSR is measured with a sampling frequency of 250 Hz.

The temperature measurements are performed through a 1-wire DS1825 digital thermometer manufactured by Maxim Integrated (San Jose, CA, USA). The 1-Wire® communication protocol implemented in the DS1825 allows the use of only one data line and ground for the communications with the microprocessor as well as for the power supply. The DS1825 incorporates a hardware 4-bit identification code that allows the connection of several temperature sensors to the same data line. The GSR measuring unit is provided with two temperature sensors, one external temperature sensor that is in contact with the skin and one internal temperature sensor placed in the microcontroller board. A sampling frequency of 1 Hz is used to measure both temperatures.

#### 2.1.2. ECG amplifier and EBI Plethysmographer

**Figure 3.**
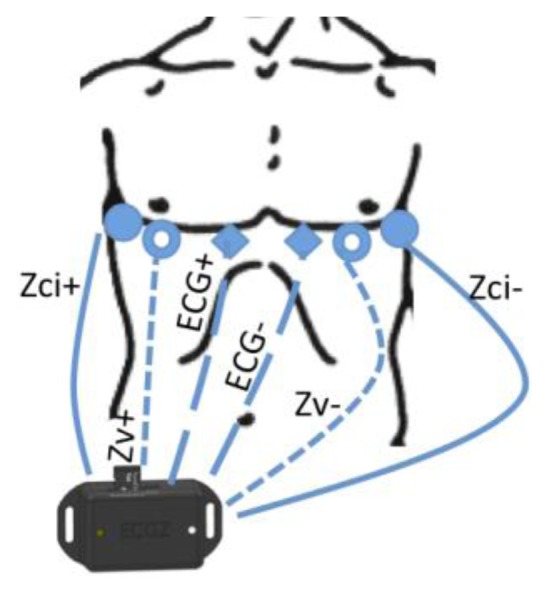
Drawing of the ECG/ICG measuring device indicating the connection with the biopotential and the TEB textrodes placed on the torso for performing ECG and respiration measurements.

This device records both the ECG biopotential signal as well as Thoracic Electrical Bioimpedance at a single frequency of 50 kHz. Its reduced dimensions (50 × 35 × 20 mm), makes the device suitable for easy integration with the chest strap electrode system described in the following section. The intended use for this ECG/EBI sensing unit is to record cardiogenic biopotentials to compute the HR from the acquired ECG and measure the impedance change caused during breathing to extract the RespR from the recorded changing TEB signal. In Figure 3 it is possible to observe the device with a typical electrode connection for ECG and TEB.

The ECG signal is measured by a 1-lead instrumentation amplifier topology suggested by Merritt *et al.* [44], the output voltage is connected to an ADC input in the microcontroller and recorded with a sampling frequency of 250 Hz. The TEB measurement is obtained using an excitation frequency of 50 kHz and a sampling frequency of 100 Hz. The impedance estimation core is based in the SOC AD5933 manufactured by Analog Devices (Norwood, MA, USA), implementing an analog front-end customized for four-electrode measurements [45].

### 2.2. Sensorized Garments and Textrodes

The following garments have been confectioned with several types of textile materials including conductive fabrics used for the textrodes and electrical connections. The conductive fabric used is the Shieldex^®^ Fabric P130+B manufactured by STATEX GmbH, (Bremen, Germany). All the sensorized garments and their different supporting elements required to operate with the textile-enabled instrumentation mentioned in the previous section are described in this section.

#### 2.2.1. Glove for GSR and Skin Temperature

**Figure 4.**
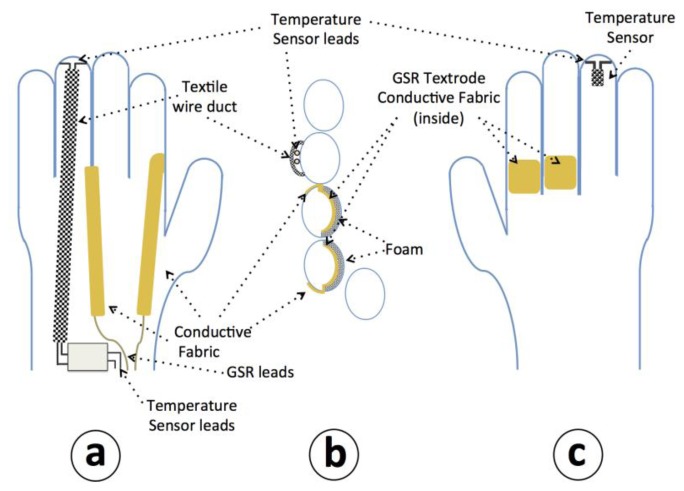
Drawing of the sensorized glove. (**a**) Upper view of the glove. (**b**) Cross-sectional view of the glove at the proximal phalanx in a perpendicular plane to the palm. (**c**) Palm view.

Two textile electrodes have been integrated inside of the glove in the proximal phalanx of the index and middle fingers on the inside of the glove for measuring the GSR, and a temperature sensor has been placed in the tip of the ring finger of the glove to sense the peripheral skin temperature, see Figure 4 for details.

**Figure 5.**
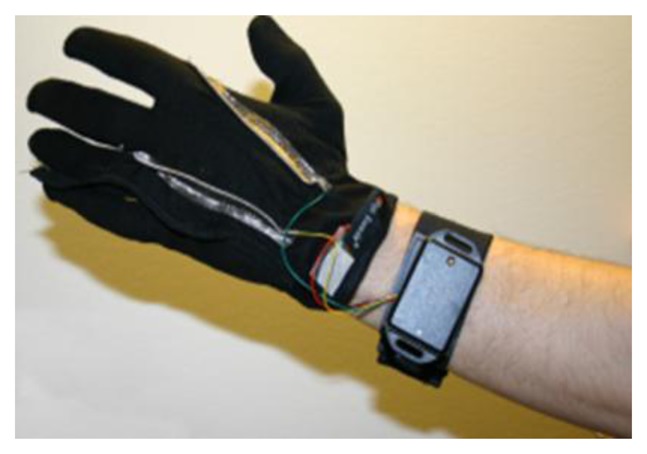
Sensorized glove connected to the measuring unit fasten to the wristband.

The textrodes and the temperature sensors are connected through 4 cables with the measuring device that is fastened with Velcro to a wristband as shown in Figure 5.

#### 2.2.2. Upper-Arm Strap for GSR and Superficial Temperature

**Figure 6.**
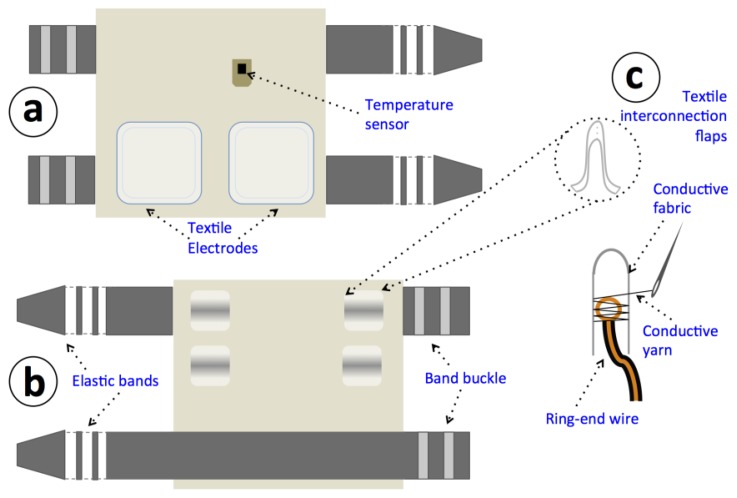
Drawing of the confectioned sensorized upper arm strap. (**a**) Inside view, showing the sensors. (**b**) Outside side, where the sensing device is placed and connected. (**c**) Detail of the textile-electronic interconnection achieved by using conductive fabrics, yarn and sewing them through a ring-shaped end.

**Figure 7.**
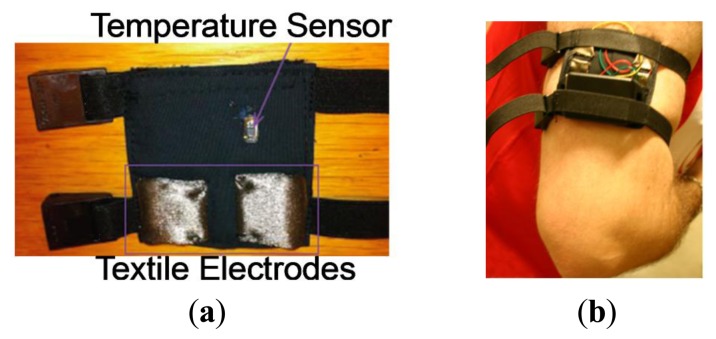
(**a**) Confectioned upper arm strap. (**b**) Measuring device and strap worn on the upper arm.

An upper arm strap has been confectioned with two integrated textrodes to sense the galvanic skin response in the skin surface. A DS1825 sensor is also integrated in the inner lining of the strap in order to contact the skin and in this way measure the skin surface temperature. Figure 6 shows the design of the confectioned sensorized garment and Figure 7a shows the confectioned sensorized upper arm strap and Figure 7b shows the same arm strap connected to the measurement unit for GSR and body temp measurement while worn on the arm.

#### 2.2.3. Chest Straps System for Cardiac and Respiration Recordings

**Figure 8.**
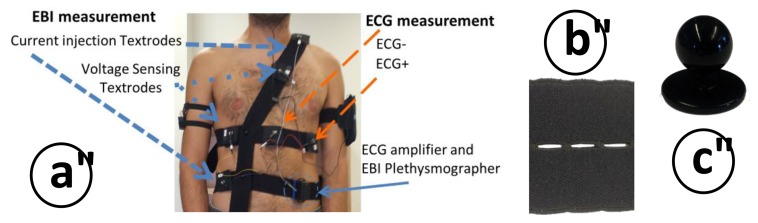
(**a**) Chest straps system confectioned for placement of ECG and TEB electrodes. (**b**) Detail of the elastic perforated band. (**c**) Fixation between straps through chef jacket button.

A chest strap garment with repositionable textile electrodes to record 1-lead ECG from two textrodes and tetrapolar TEB measurements with another four textrodes has been developed. The possibility of placing the electrodes in any place along the horizontal and vertical straps enables to test different measurement configurations for ECG and TEB measurements, see Figure 8a. Depending on the placement of the textrodes around the surface of the thorax and abdomen, the TEB measurement will have more or less cardiac and respiratory components allowing us to perform a multi-parametric signal recording if required.

Each strap is made with a highly elastic band with 1 cm perforations every 2 mm, see Figure 8b. These perforations enable the reposition of the textrodes along the straps and fixation between straps using chef jacket buttons, see Figure 8c.

#### 2.2.4. Repositionable Textrodes

**Figure 9.**
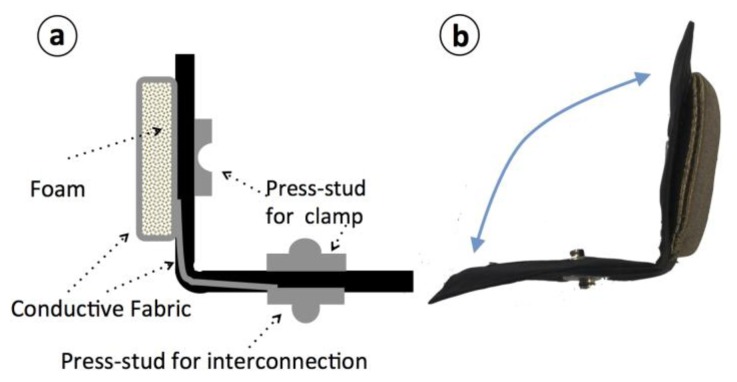
(**a**) Schematic of the repositionable textrode. Notice that when folded the electrode is clamped using its male and female press-studs, thus creating an electrical and mechanical connection. (**b**) Confectioned repositionable textrode.

The textile electrodes have an approximate surface of 25 cm^2^ and are confectioned with a textile structure that is folded over itself creating a clamp through a male-female pair of press-studs, as shown in Figure 9. Note that the connection between the measurement leads and the textrode is achieved through a third press-stud, male.

### 2.3. Voice Recording

In order to record the voice during the experiments of the first phase of the project, a smartphone was custom programmed for such purpose. The selected device was the BRVAR909 manufactured by Bravus Europe AMISL (Andorra), an Android 2.2 based rugger device, IP67 specifications complaint including water and shock resistant. An 8 GB SD card was used as main storage system.

A customized Android application was designed and programmed, to allow the synchronized recording of the speakers' speech at 22.5 kHz. The local clock of the smartphone was also registered in this way, once the clock was synchronized with the rest of the measurement devices, the recordings of the speech could use the same time frames.

## 3. Methods

### 3.1. Activities for Assessment of Stress

In order to evaluate the performance of the prototype designed in the first phase of the project, an experiment was designed with the purpose of generating different types of stress in a set of subjects. The main purpose of this experiment was the controlled generation of emotional, mental and physical stress, through a set of stages. A PC-based application was created to induce stress situations in a controlled manner. The main tools used to generate stress are described in the following sections for each different type of stress.

#### 3.1.1. Emotional Stress

In order to generate emotional stress, a set of different videos was used. The videos used were selected to specifically generate several types of emotions, including neutral, sadness, anger and disgust. In a first step, the videos were viewed without the interaction of the subject, and in a second step, they were repeated and the subject had to click under the emotion perceived in any moment. For this purpose, a circle of emotions inspired in the Plutchick circle was used [46]. Each video used in the experiment was based on a set of segments of movies with a total duration of approximated 10 min, and the movies used were Earth (2007), Life is Beautiful (1997) and Cannibal Holocaust (1980).

#### 3.1.2. Mental Stress

In a different way, mental stress was generated through two different games. These games were selected in order to put the subject at the limits of mental calculus. The first game consists in making a set of quick mental sums. A set of digits to be added appears on the screen, and the subject has to click on the correct solution. The time available to solve each problem varies along the experiment, being decreased when the subject answers right, and increased when he/she answers wrong. The idea is to put the subject in a high level of mental activity, independently of his/her calculus ability. This game was played since 80 sums from two to five digits were carried out. The second game consists in a Tetris-based game, in which again the objective is to seek the limit of the mental ability of the subject. For this purpose, the speed of the game was controlled so the difficulty could be adapted during the exercise.

#### 3.1.3. Physical Stress

Finally, physical stress was generated making the subject go up and down in the main stairs of a three level building during 5 min. This activity was selected due to its simplicity and since it quickly generates a high physical stress level.

#### 3.1.4. Surveys and Questionnaires for Stress Assessment

Both socio-demographical and psychological computer-based surveys were included in the experiments. The socio-demographical survey aimed at registering all the relevant information from each subject, including personal data, main biometrics, sport activities, any toxic habits and details about what the subject did during the hour previous to the experiment.

Concerning the psychological surveys, their purpose was to monitor the stress level and typology during the different parts of the experiments. The Self-Assessment Manikin [47] and the Profile of Mood States [48] were carried out after the visualization of each video in the emotional stress stages.

Short computer-based control surveys were also carried out after every stage of the experiment, in order to determine if the subject was concentrated on each task or if, by the contrary, he/she got bored during the experiment. The aim was to determine the effectiveness of the stress situations along the experiment.

#### 3.1.5. Baseline and Sequence of Experimental Setup

The total duration of the experiment was around 75 min. In a first step after starting the recording of the different devices of the prototype, the subject fills out a sociocultural questionnaire. The aim is to establish a baseline in the register. Once the sociocultural questionnaire is completed, the different stages of the experiment are carried out. It is important to highlight that several surveys are also carried out in order to monitor the real stress level of the subject during the experimental process. The sequence of the experiment is described as follows:

(1). Sociocultural survey.
(2). Neutral stage: neutral video.
(3). First mental stage: sum game.
(4). First emotional stage: sad video.
(5). Second mental stage: Tetris game.
(6). Second emotional stage: disgusting video.
(7). Physical stage: stair running.

### 3.2. Operational Test of the Wearable Biomedical Measurement Devices and Sensorized Garments

A total number of 12 measurement devices and sensorized garments have been produced for each modality. The correct operation of the measuring devices and the corresponding sensorized garments has been validated at least on two different healthy volunteers obtaining at least 2 min of GSR recordings with the glove and the arm strap as well as ECG and respiration activity. In addition, to such test performed systematically prior to the initiation of each measurement session of 75 min, non-systematic test have been carried out all through the implementation of the project. e.g., battery life test, where the systems were used continuously for more than 2 h.

### 3.3. Speech Analysis

Voice was recorded during the experiments with the aim of studying its use in order to assess emotional status. Several features were successfully calculated from the speech recordings, with the aim of implementing those more useful to determine the stress level. The features extracted in the voice analysis were statistical parameters from Mel Frequency Cepstral Coefficients, short time energy, pitch, pitch perturbation quotient, amplitude perturbation quotient, jitter, voice turbulence index, and soft phonation index.

### 3.4. Experimental Recordings Performed with the Wearable Biomedical Measurement Systems

Non-invasive recordings have been carried out with the 12 sets of wearable measurement devices and sensorized garments in 42 volunteers undergoing the battery of stress test described in previous sections. This way over 3,000 min of multichannel recordings have been produced.

### 3.5. Analysis of Multi-Sensorial Data

A total of 724 features from seven different signals were used to extract the information derived from the stress levels of the subjects. Time-Frequency and Statistical analysis has been performed on each of the different type of the signals. Several time configurations were tested in order to determine the best time frames for the stress analysis. For this purpose, two parameters were taken into account: the frame length and the frame overlap. Frame lengths of 10, 30, 60, 180 and 300 s were evaluated, and time overlaps of 0, 10, 30 and 60 were considered. From the preliminary results, we selected a frame length of 60 s and a time overlap of 10 s, so that a decision was taken every 10 s using information from the last 60 s of each signal. These values represented a tradeoff between time resolution and performance.

## 4. Results

### 4.1. Validation of Sensorized and Wearable Biomedical Measurements Systems

This section presents the measurements recorded with the measuring devices described and the sensorized garments in Sections 2.1 and 2.2, respectively. More specifically, this section presents a set of measurements obtained during the validation test with the one measurement device and garment of each kind for a single subject. More detailed information has been presented in [42].

#### 4.1.1. GSR and Temperature

**Figure 10.**
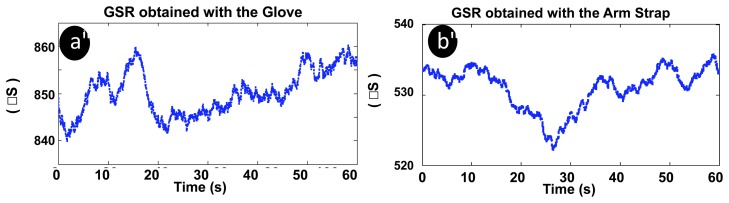
Galvanic skin response measurements. (**a**) Glove. (**b**) Upper arm strap.

Skin temperature and GSR were recorded in several subjects. While skin temperatures produced very stable measurements around 34 °C with both garments producing flat recordings, GSR measurements exhibited larger variance, see Figure 10. Note that the GSR data has been filtered with a moving average window of 5 s as in [49].

#### 4.1.2. ECG Recordings

**Figure 11.**
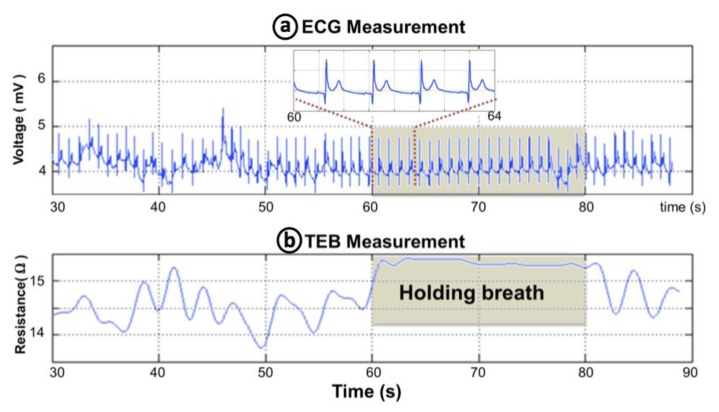
(**a**) ECG recording. (**b**) Transthoracic Electrical Bioimpedance recording.

Surface cardiac biopotentials were recorded in several subjects with the wearable measurement system presented in Figure 8a. The ECG recording plotted in Figure 11a shows 60 s of an ECG record. As shown in the 4 second-long zoomed segment the recording is very fair, allowing for a straight detection of the R complex for heart rate assessment. The first 25–30 s show slow activity related with respiratory movement. In the plot it is possible to see that during the seconds 60 to 80 while the subject kept the breath, the ECG recording was completely stable.

#### 4.1.3. Thoracic EBI Recordings

TEB recordings were obtained simultaneously to the ECG recording, see Figure 11, using the wearable measurement system presented in Figure 8a for several subjects. A 60-second segment of the TEB recording is shown in Figure 11b. The respiration activity is easily observable in this plot as the resistance increases and decreases according to the respiration cycle. Note that the changes stop between 60 and 80 s on the plot while the subject was holding his or her breath.

### 4.2. Voice Recordings

**Figure 12.**
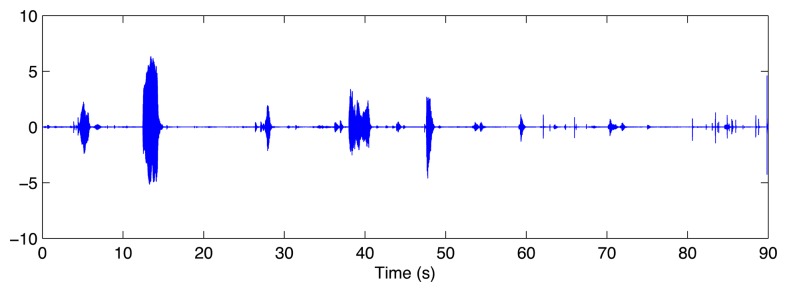
90 s speech recording obtained with the BRVAR909 smartphone for one of the subjects of the experiments.

The recordings obtained with the BRVAR909 presented clear speech signals as shown in Figure 12, where a segment of 90 min is shown.

### 4.3. Measurement Database

The experimental test produced full recordings for 42 subjects from February 2013 to May 2013; generating more than 50 h of multisensorial data, see Table 1. All signals were stored in raw data, and later processed to extract features potentially useful to implement detection algorithms and classifiers for assessment of the user's mental stress.

### 4.4. Preliminary Analysis of Acquired Data

It is important to recall that the aim of the experiments during the first phase was to establish the potential usefulness of the different type of recordings measured with the implemented prototypes. Notice that, for such analysis work, the database was split in two: the first 25 subjects were used for design and training, and the remaining 17 subjects were used to testing purposes to validate the performance of the classification algorithms.

For this purpose, a 4-class classification scenario was designed, with the aim of determining in which stage was the subject: neutral stage, emotional stress stage, mental stress stage and physical stress stage. As previously indicated, a time window with a length of 60 s and a time overlap of 10 s was used, *i.e*., a decision was produced every 10 s using data from the last 60 s of each recording. These values represented a tradeoff between time resolution and performance.

From the 724 evaluated 192 were selected using a genetic algorithm [50] that were run through a Linear Discriminant Classifier (LDC).

Table 2 shows the results obtained with the implemented classifiers. Both the number of features selected by the genetic algorithm and the error rate obtained by the LDC are included. Results show the best performance of the electrocardiogram signals and the thoracic impedance signals, when compared to the other signals recorded in the experiments. The 10 features selected from the ECG recording and the 20 selected from the TEB measurement are enumerated in two lists in Appendix, respectively. It is important to highlight the performance of the speech analysis that resulted in a very high error rate. This is mainly caused by the absence of voice in most of the recorded data, making the speech-based classifier to achieve higher error rates.

## 5. Discussion

### 5.1. Wearable Biomedical Measurement Devices and Sensorized Garments

Small sized measurement devices were made specifically to facilitate the integration with the sensorized garments. State-of-the-art techniques were used for textile-electronic integration like press-studs, which pressed through a conductive fabric is the most used approach to perform such type of textile-electronic interconnections and it can be found in commercial products [5,7] as well as in research prototypes [51,52]. Sewing with conductive yarn through conductive fabrics has been used before to establish an electrical connection [53]. The sensorized garments have fulfill their intended purpose in Phase 1 allowing the acquisition of several different physiological measurements during exercises designed to specifically target assessment of stress. The performance of the measurement devices and the sensorized garments was previously validated in [42,43].

### 5.2. Preliminary Observations from Stress Experiments

It is clearly noticed from the error rate reported in Table 2, that the biosignals producing the best classification of mental status are the ECG, mainly heart rate and the respiration rate. Such results may be expected since both respiration and heart rate are highly mediated by the ANS and also it is relatively easier to obtain robust ECG and TEB measurements with tight garments on the thorax than GSR or temperature from fingers or arms. A detail description of the multiparametric and classification work performed on the database will be reported elsewhere.

The obtained error rates in detection of stress from heart rate and the respiration rate, 23.72% and 24.40%, respectively, are slightly better than 31% error rate obtained from HR variability analysis as shown in [54]. The performance exhibit by heart rate and the respiration rate is also superior than the performance obtained from analysis of the electroculogram with an error rate >25% [55], while they are comparable to the performance obtained from ECG analysis [55] and significantly worse than the performance obtained from EEG analysis, with error rates as low as 6% [55].

### 5.3. Voice Recording

The classification error obtained with the analysis of the recorded speech (>48%) is slightly over what other authors have reported in [56] for automatic classification of stress from speech analysis (46% & 44%, using Hidden Markov Models and Vector Quantification, respectively). When comparing the 48% error rate obtained in this study with the error rates obtained with traditional Teager Energy Operator-based feature vector with a Hidden Markov Model-trained classifier (22.5% error), or using a weighted sub-band detection scheme (4.7%) [57] the differences are significant.

In either case the error rate obtained based on ECG or TEB features was significantly lower. The relatively larger error rate produced when using speech-based features might be due to the fact that while voice recordings were performed in every experiment for the duration of the experiment, unfortunately the test activities did not produce enough speech content to be able to characterize the different states properly. Hopefully during the final validation test planned for the end of the second phase, the voice recordings will contain more speech information useful for classification purpose.

### 5.4. Lessons Learned

Executing this project has given us the opportunity to obtain new experiences and learn from them. One issue that we find important is judging what is enough, otherwise the production of excess data, samples or data might increase the requirements of the project unnecessarily and can have undesired consequences. Considering the exploratory nature of the first phase, manufacturing 12 different sets of measurement devices and sensorized garments was completely unnecessary and not efficient since it required more financial and staff resources. The fact is that the experiments were never done simultaneously, which means that a few measurement sets would have been enough.

Another lesson learnt that had a significant influence on the realization of the experiments was the use of a SD card for data storage and also for data access, instead than an alternative method for data access like Bluetooth. The lack of this feature meant that prior to the execution of each experiment to perform a operational test, the SD card had to be removed and read in the PC and despite this fact, there were always doubts about whether the measurements were being recorded properly because the access to the recorded data could not be done until the experiment was finalized. This limitation has been overcome in the measurement device implemented during Phase 2.

## 6. Conclusions and Outlook

The wearable measurement systems and the voice recording system have allowed the execution of activities specifically targeted to evaluate the influence of stress on several physiological variables. Therefore the 1st phase of the ATREC project has been completed and the preliminary analysis from the experimental results suggest that for the 2nd phase, it is very likely that a sensorized vest or T-shirt with integrated textrodes for biopotential recordings and trans-thoracic bioimpedance measurements will be manufactured, similar to the one reported in [58].

## Figures and Tables

**Table 1.** Signals recorded on the database.

**Signal**	**Site or Configuration**	
Electrocardiogram	In thoracic region, in an axial plane	
Thoracic Electrical Bioimpedance	Transthoracic Respiration	
Galvanic Skin Response	Between Middle and Index Fingers	Back of Upper Arm
Temperature	Tip of Ring Finger	Back of Upper Arm
	Ambient Temperature	
Speech	Built-in Microphone on the Smartphone	

**Table 2.** Signals recorded on the database.

**Signal**	**Nr of Extracted Features**	**Selected Features**	**Error Rate**
Electrocardiogram	244	10	23.72%
Thoracic Electrical Bioimpedance	244	20	24.40%
Temperature (Arm)	16	7	44.53%
Speech	44	15	48.44%
Galvanic Skin Response (Fingers)	80	40	48.48%
Temperature (Finger)	16	4	56.80%
Galvanic Skin Response (Arm)	80	20	57.85%

Note: Ordered according to classification performance.

## Appendix

### Features Selected for the Obtained Error Rates

#### A1. From Electrocardiogram

Mean value of the heart rate

- Standard deviation of the heart rate
- Third quartile of the heart rate
- Logarithm of the spectrum in mid frequencies (0.08 Hz–0.15 Hz) of the heart rate
- Ratio of the logarithm of the low frequency energy (0.04 Hz–0.08 Hz) *vs.* Logarithm of the high frequency energy (0.15 Hz–0.5 Hz) of the heart rate
- First quartile of the heart rate variability
- Harmonic mean of the heart rate variability
- Logarithm of the standard deviation of the heart rate variability
- Average number of consecutive RR intervals separated more than 50 ms (pNN50)
- Mean value of the ultra-low frequency band (0 Hz–0.04 Hz) of the heart rate

#### A2. From Thoracic Electrical

- First quartile of the low frequency component (0.04Hz–0.08Hz) of the TEB
- Mode of the mid frequency component (0.08 Hz–0.15 Hz) of the TEB
- Minimum value of the high frequency component (0.15 Hz–0.5 Hz) of the TEB
- Mean value of the ultra-low frequency band (0 Hz–0.04 Hz) of the TEB
- Mean value of the respiratory rate
- Standard deviation of the respiratory rate
- Third quartile of the respiratory rate
- Kurtosis coefficient of the respiratory rate
- Sample entropy of the second differentiation of the respiratory rate
- Sample entropy of the third differentiation of the respiratory rate
- Variance coefficient of the inspired respiratory flux
- Mean value of the total respiratory time
- Standard deviation of the total inspiration time
- Harmonic mean of the respiratory rate
- Trimmed mean of the total respiratory time
- First LPC coefficient of the total respiratory volume
- Mean value of the inspired respiratory flux
- Variation coefficient of the inspired respiratory flux
- Mean value of the ultra low frequency band (0–0.04 Hz) of the inspiration time
- Mean value of the ultra low frequency band (0–0.04 Hz) of the total respiratory volume
